# Photoacoustic imaging for monitoring of stroke diseases: A review

**DOI:** 10.1016/j.pacs.2021.100287

**Published:** 2021-07-24

**Authors:** Xi Yang, Yun-Hsuan Chen, Fen Xia, Mohamad Sawan

**Affiliations:** a Zhejiang University, Hangzhou, 310024, Zhejiang, China; b CenBRAIN Lab., School of Engineering, Westlake University, Hangzhou, 310024, Zhejiang, China; c Institute of Advanced Technology, Westlake Institute for Advanced Study, Hangzhou, 310024, Zhejiang, China

**Keywords:** Photoacoustic imaging, Optoacoustic imaging, Biomedical imaging, Stroke, Cerebrovascular imaging

## Abstract

Stroke is the leading cause of death and disability after ischemic heart disease. However, there is lacking a non-invasive long-time monitoring technique for stroke diagnosis and therapy. The photoacoustic imaging approach reconstructs images of an object based on the energy excitation by optical absorption and its conversion to acoustic waves, due to corresponding thermoelastic expansion, which has optical resolution and acoustic propagation. This emerging functional imaging method is a non-invasive technique. Due to its precision, this method is particularly attractive for stroke monitoring purpose. In this paper, we review the achievements of this technology and its applications on stroke, as well as the development status in both animal and human applications. Also, various photoacoustic systems and multi-modality photoacoustic imaging are introduced as for potential clinical applications. Finally, the challenges of photoacoustic imaging for monitoring stroke are discussed.

## Highlights

• Summarizes photoacoustic imaging for monitoring of stroke diseases.
• Focuses on the potential photoacoustic systems for human monitoring.
• Reports the challenges of stroke monitoring in the human brain.

## 1 Introduction

Stroke is the leading cause of death and disability after ischemic heart disease [1,2]. China has the largest age-standardized incidence of stroke (3.54 strokes per thousand person-years) in the world. According to the causes, strokes are classified into ischemic and hemorrhagic strokes. From the 2016 Global Burden of Disease (GBD) study, ischemic stroke is the main type of prevalent stroke, accounting for 84.4 % of the prevalent strokes [1]. Patients who suffer from stroke diseases usually have other chronic diseases like hypertension, diabetes, atrial fibrillation, and atherosclerosis [1,3]. Unhealthy lifestyles including smoking and drinking are associated with the risk of stroke occurrence [3]. Monitoring or detecting the indexes related to stroke diseases is important for the prediction and prevention of stroke diseases in the early stage.

Besides, monitoring the cerebrovascular system is important for the diagnosis and treatment of stroke disease. At present, X-ray computed tomography (CT) and magnetic resonance imaging (MRI) have been widely used for stroke diagnosis and treatment periods clinically [[4], [5], [6]]. However, due to the ionizing radiation of the CT, the method cannot be used several times within a short period. MRI is low temporal resolution and also limited in the condition of the metal materials. Therefore, it is difficult to continually monitor the status of blood flow. During the diagnosis and the treatment, physicians need to have knowledge and experience to make the decision. Once blood flow in the brain can be monitored for a long time, it would increase the accuracy of treatment and reduce the damage due to stroke.

For better prediction, diagnosis, and treatment of stroke, a safe imaging method with a high spatial and temporal resolution is needed. With the improvement in optical and ultrasonic devices, photoacoustic imaging (PAI) being a non-invasive, label-free, functional imaging method with high resolution has been developed recently. It presents a potential alternative for high-accuracy monitoring of stroke diseases. Therefore, the potential of the PAI for stroke disease monitoring is reviewed in the following sections. We illustrate the stroke diseases and the present imaging methods for stroke including X-ray computed tomography (X-ray CT), magnetic resonance imaging (MRI), positron emission tomography (PET), single-photon emission computed tomography (SPECT), ultrasound imaging, optical imaging, electroencephalography (EEG), and functional near-infrared spectroscopy (fNIRS) in section 2. Then, the PAI technique and its application to stroke diseases are introduced in section 3. Specifically, we discuss the progress of PAI for stroke and related diseases, the status from small animals to the human brain, as well as the PAI system and multi-modality imaging. Finally, the challenges and prospects for stroke imaging in the human brain also are discussed in section 4.

## 2 Stroke imaging

In this section, we introduce the stroke causes to understand the mechanism of the stroke occurrence with different causes and risks. Then, the main clinical and available imaging methods for stroke monitoring and detection have been discussed and compared.

### 2.1 Stroke causes

There are two main causes of stroke, ischemic stroke, and hemorrhagic stroke. Ischemic stroke is generally caused by cerebral ischemia due to blood vessel blockage caused by blood vessel stenosis and thrombosis. Some vascular stenosis is caused by deformity or lack of elasticity during the growth of blood vessels, while others are caused by the thickening of the vessel wall due to matter deposition. Common diseases such as atherosclerosis are led by the deposition of lipids in the blood vessel wall [7]. Metabolites and blood cells in the blood vessels make the vessel narrow, and then gradually block the blood vessels. Hemorrhagic stroke is caused by bleeding due to the damage of the blood vessel or blood-brain barrier. The damage of the blood vessel or blood brain barrier can be resulted from sudden changes of the blood pressure. Sometimes bleeding may be caused by aneurysms [8] or cerebral arteriovenous malformations in the brain [9].

No matter it is an ischemic or hemorrhagic stroke, it will cause brain and blood metabolism problems and make abnormal brain function. The patients suffered from the stroke can suffer from functional impairment or death. Usually, the symptom of the stroke can occur quickly after the stroke happens. Once the symptom disappears after 24 h, the stroke is named transient ischemic attack (TIA) [10]. If the treatment of the stroke is not in time, the patients may have permanent sequelae. During the diagnosis and therapy of the stroke, biomedical imaging for the blood vessel, brain lesion, and other medical examinations will be conducted to find the risk factors that lead to stroke and eliminate other possible causes [11].

### 2.2 Imaging modalities for stroke

To track the progress of a stroke, it is necessary to characterize the cerebrovascular system at various stages of the stroke. Before the onset of stroke, predicting the risk of stroke based on the images can avoid its occurrence and reduce the loss or damage of neuron cells. After the onset of stroke, images of the cerebrovascular condition provide information for clinical physicians to evaluate its severity and to determine the therapeutic method or dosage. Moreover, the imaging techniques evaluate the progress of recovery after therapy. The imaging techniques with high sensitivity to the objects inside the vascular system, such as hemoglobin, lipids, and tumors, are required. To be suitable for long-term monitoring of the cerebrovascular system, the system must be safe, noninvasive, label-free, and portable. For better prediction, diagnosis, and treatment of stroke, a safe imaging method with a high spatial and temporal resolution is needed.

The advantages and drawbacks of various imaging techniques available for stroke are compared in Table 1. X-ray CT is the first widely used imaging test to identify lesions for disease diagnosis which can be combined with PET and SPECT [12,13] to increase specificity and performances of functional imaging. However, these methods utilize ionizing radiation to obtain images inside the body, resulting in safety issues when frequently used. To avoid the impact of ionizing radiation, functional MRI (fMRI) is used for clinical diagnosis as a non-ionizing method. The main limitation of fMRI is low temporal resolution [14]. Besides, the X-ray CT and MRI equipments are bulky and inconvenient for in-time monitoring. Thus, conventional devices such as CT, PET, SPECT, and MRI, are unsuitable for long-term stroke management meaning monitoring cerebrovascular events of the brain. Optical coherence tomography (OCT) is available for detecting blood vessels with high specificity, and high and temporal resolutions. But this non-invasive method just works on small animals due to the limited optical propagation [15]. Ultrasound imaging has been used for carotid artery imaging to provide anatomical and hemodynamic information and analyze the causes of the stroke. But this method has low specificity of the tissue. Notably, the higher sensitivity of the vascular system and deeper imaging in the brain must be considered simultaneously. Other techniques to study the mechanism of stroke include EEG and fNIRS [16,17], which are wearable systems for long time monitoring. However, they are limited in a spatial resolution at centimeters range and detection depth below the cortical surface. Among the available imaging techniques, PAI has the potential to fulfill the requirements for stroke monitoring clinically with high resolution and propagation depth for quantitative measurement in the human brain.

**Table 1:** Comparison of various imaging techniques for stroke. Adapted from [[18], [19], [20], [21]].

Techniques	Imaging mechanism	Spatial resolution	Imaging depth	Advantages	Disadvantages
X-ray CT	X-ray absorption	High	Whole-brain	• Nondestructive • Superior resolution for microvascular imaging • High signal to noise ratio	• Ionizing radiation • Low soft-tissue contrast • Exogenous contrast agents for blood vessels and flow • Isolated room to store the equipment
PET/ SPECT	Positron/ photon annihilation	High	Whole-brain	• Functional information • High detection sensitivity • Molecular imaging	• Ionizing radiation • Lack of structural information • Exogenous radiolabels • Poor spatial resolution • Longer scan times • Low signal to noise ratio • Nonspecific uptake of radiotracers
MRI	Nuclear magnetic resonance	High	Whole-brain	• No radiation • No intra-arterial puncture • Excellent soft-tissue contrast • Superior tissue differentiation • Anatomical, functional, and molecular information	• High-intensity magnetic field • The spatial resolution is inversely proportional to the field of view • Low temporal resolution • Metal influence • Huge size of the equipment
US	Ultrasound scattering	Average	Centimeters in soft tissue	• Non-ionizing • Superior temporal resolution • Anatomical, mechanical, and functional information	• Anisotropic • Limited soft-tissue contrast • Acoustic artifacts • Difficult to interpret
OCT	Light scattering	High	<3 mm in soft tissue	• Non-ionizing • Real-time imaging • High spatial and temporal resolution • High sensitivity • Label-free imaging	• Limited imaging depth • Susceptibility to photobleaching • Limited *in vivo* imaging
PAI	Light absorption	High to average	Below cortical surface	• Non-ionizing • Real-time imaging • High imaging depth with a reasonable spatial resolution • Label-free imaging of blood vessels • Functional information • Direct blood metabolism information	• Lack of anatomical information • Susceptible to acoustic artifacts • Spatial resolution is inversely proportional to the propagation depth
EEG	Electroencephalography of brain activity	Low	Below cortical surface	• No radiation • Functional imaging • High temporal resolution • Long time monitoring • Direct brain activity information	• Time-consuming of the cap preparation due to the electrode positioning • Low spatial resolution • Indirect blood metabolism information
fNIRS	Light reflectance	Low	Below cortical surface	• Non-ionizing • Functional imaging • High temporal resolution • Long time monitoring • Direct blood metabolism information	• Time-consuming of the cap preparation due to the detectors/emitters positioning • Low spatial resolution

X-ray CT, X-ray computed tomography; PET/SPECT, positron emission tomography/single-photon emission computed tomography; MRI, magnetic resonance imaging; US, ultrasound; OCT, optical coherence tomography; PAI, photoacoustic imaging; EEG, electroencephalography; fNIRS, functional near-infrared spectroscopy.

## 3 Photoacoustic imaging for stroke

Photoacoustic Imaging, an important potential alternative to present clinical methods such as CT and MRI, has significant advantages for label-free dynamic imaging. Stroke is a disease related to changes in vascular conditions resulting from the physiological activity. Therefore, the PAI technique is expected to apply on the stroke monitoring. In this section, we describe the principle and characteristics of PAI, the research progress about stroke and related disease applications. Also, status from small animals to humans, PAI system, and multi-modality imaging are covered.

### 3.1 Introduction to PAI

**Fig. 1:**
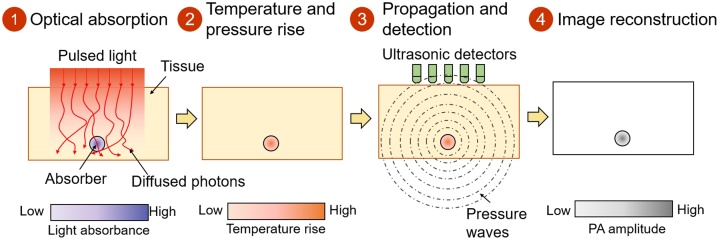
Schematic diagram of photoacoustic imaging principle: (1) With pulsed light applied to an object’s site, tissues absorb the light’s energy; (2) The absorbed light energy is converted to heat energy which increases the transient temperature, then the surrounding pressure increases due to thermoelastic expansion; (4) Pressure propagates as photoacoustic waves from the site of interest toward the surface and are acquired by ultrasound detectors; and (5) Propagating signals are reconstructed into images.

The photoacoustic effect was found by Alexander G. Bell in 1880 [22]. Based on the photoacoustic (PA) effect, PAI has been researched for decades with the development of lasers, ultrasound transducers, and computers. Fig. 1 shows the principle of PAI. Typically, the PA effect begins when a pulsed light is directed at the target tissue. The energy of the propagating photons is absorbed by the molecules inside the tissue, which excites the status of the absorbers from the ground state to the excited state. When the matter returns to the ground state, the energy is converted into heat, which increases the transient temperature. The pressure inside the tissue increases owing to thermoelastic expansion. The pressure propagates as photoacoustic waves, which are detected by ultrasound sensors. Finally, PA images are obtained through computer computation based on the signal process and image reconstruction.

The PAI technology, which combines the advantages of both optics and ultrasound, is a relatively novel method for medical imaging without ionizing radiation. Compared with the traditional optical imaging methods, the photoacoustic method overcomes the obstacle of light scattering through the biological tissue. Owing to the variations of the photoacoustic effect in different biological tissues, the optical absorption by those tissues results in high contrast between them. In addition, the photoacoustic wave generated by a deep tissue can be detected by an ultrasound transducer without mutual interference of transmission and degradation of the signal quality. With the advantages of combined optical specificity and acoustic penetration, PAI was introduced to bridge the gaps between the resolution and penetration depth in brain imaging.

PAI has high specificity for intrinsic biological objects which can be used for many disease diagnoses. Biological molecules includes oxyhemoglobin (HbO_2_) [23,24], deoxyhemoglobin (HbR) [25,26], melanin [[27], [28], [29]], lipids [[30], [31], [32]], proteins [33], nucleic acids [34] [35] and carbohydrates [36]. The objects can be detected using different optical wavelengths owing to their different absorption coefficients along various optical wavelengths. The specific wavelength is selected to identify the object based on the absorption peaks or other characteristic points [37]. The green light of 532 nm as the isosbestic wavelength of the two types of hemoglobin is often used for imaging hemoglobin [38]. Sometimes HbO_2_ and HbR are distinguished by lasers at two different pulse rates, such as 2 ns and 2 ps [39]. In addition, applying one isosbestic wavelength at 532 nm and another non-isosbestic wavelength at 560 nm is another approach to obtain the images of hemoglobin (Hb) [38]. Owing to the high specificity of the photoacoustic effect in hemoglobin, PAI technology is widely explored in imaging various vasculature problems.

With the development of the PAI technology, high-quality anatomical, hemodynamic, metabolomic, and functional imaging has been obtained from the bench to the preclinical stage. Based on the photoacoustic effect, various modalities of the PAI technique have been developed for specific applications and conditions. Considering the requirements for stroke imaging, photoacoustic microscopy (PAM) for microvasculature and photoacoustic computed tomography (PACT) for large-scale imaging are highlighted. PAM is widely used to explore the details inside tissues. Based on the density of the laser focus and acoustic focus, PAM is classified into optical-resolution PAM (OR-PAM) [40,41] and acoustic-resolution PAM (AR-PAM) [42,43]. In OR-PAM, the laser focus is much tighter than the acoustic focus, which enables microscopic imaging in the brain [44], eyes [[45], [46], [47], [48]], and ears [49] of animals. In contrast, when light is less focused than the acoustic focus, it facilitates mesoscopic imaging such as the vascular shape of the skin. In addition, the axial resolution of both PAM types is determined by the acoustic properties, meaning the center frequency and bandwidth of the acoustics. A larger central frequency and wider bandwidth result in better resolution but shallower penetration. Furthermore, the lateral resolution depends on the optical and acoustic properties. To overcome the limitations of optical diffusion, super-resolution PAM (SR-PAM) [50] has been developed based on the nonlinear mechanisms of the photoacoustic effect.

Photoacoustic microscopy acquires one image based on single-transducer mechanical scanning. To obtain a image with a large field of view, PACT is a modality of PAI, which uses wide-field illumination and detects the resultant photoacoustic waves at multiple spatial positions [[51], [52], [53]]. Then, inverse modeling is employed to reconstruct the images. To optimize the performance of signal reception, the geometrical shapes of the ultrasound transducer arrays can be linear, planar, circular, spherical, and partly spherical. To increase the speed of the imaging process and balance the cost and throughput, a novel PAT based on an ergodic relay (PATER) [54], which is a low-cost, high-throughput, wide-field snapshot, has been developed. With high-velocity quantification and super-resolution imaging, PATER is promising as a portable device to monitor human signals. The ergodic relay is also investigated in multifocal PAM (MF-PAM) with a single transducer. MF-PAM detects photoacoustic signals generated from 400 optical foci at one time [55]. At present, several research groups are focusing to improve the PAI technique and its validation in various applications [[54], [55], [56], [57], [58], [59], [60], [61]]. Attention is being paid to component design and system optimization for multiscale PAI. The ultimate goal is to achieve a high-sensitivity, high-resolution, and high-depth dynamic imaging technique based on different applications.

### 3.2 Progress of PAI for stroke

In the early 2010s, researchers already proposed the potential application of the PAI in stroke diseases due to its high sensitivity and good penetration [62]. So far, researches about stroke diseases have been applied at the preclinical stage. The challenges and difficulties of the PAI in brain imaging remain, but the clinical prospect is promising. Considering the research achievements, this section introduces the research progress of the PAI mainly focusing on small animals. These studies are fundamental to further applying PAI on human subjects.

**Fig. 2:**
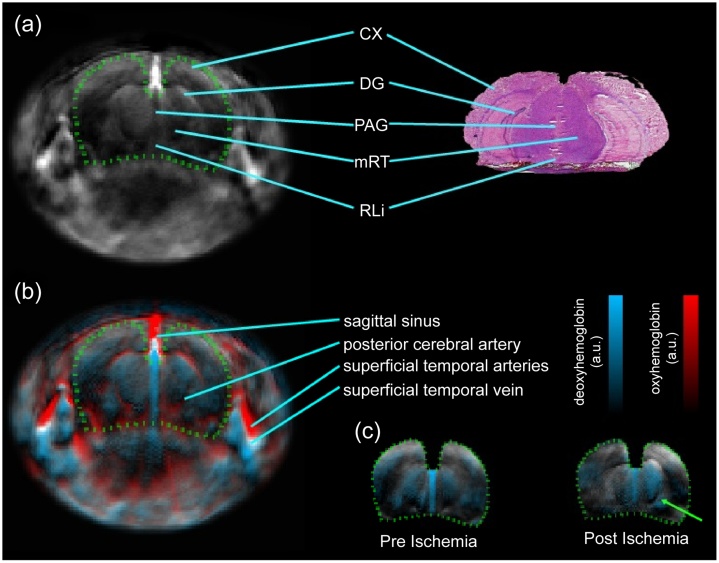
Images of the stroke mouse model: (a) Structural features of the infarct area in the photoacoustic image (left) are compared with the features in the cryosection image (right); (b) Multi-parametric photoacoustic image of unmixed oxygenated and deoxygenated hemoglobin overlaid with a 790 nm wavelength image (black) is represented by red (HbO_2_) and blue (HbR), respectively; (c) Deoxygenated hemoglobin images of two stages overlaid with the 790 nm wavelength image in the coronal slice. The area matched with the brain is depicted by the green dotted line. The green arrow shows the clear asymmetry in post-ischemia when compared with the symmetrical image at pre-ischemia. CX, cortex; DG, dentate gyrus; PAG, periaqueductal gray; mRt, midbrain reticular formation; RLi, rostral linear nucleus [65].

**Fig. 3:**
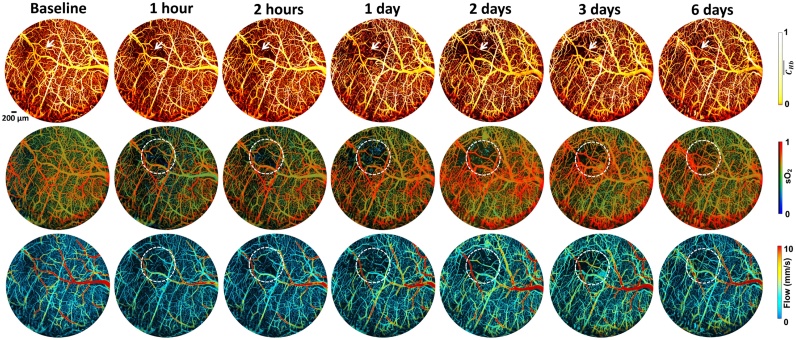
Dynamic images of the hemoglobin concentration (C_Hb_), blood oxygenation (sO_2_), and cerebral blood flow (CBF) using multi-parametric micro-level PAI. The images were obtained from a mouse cerebral arteriole with photothrombotic occlusion. The images acquired 1 h after the onset of the stroke show that the blood perfusion decreases and gradually restores after 1 day. In the dashed white circles, the increased sO_2_ with recovery capillary density and blood flow implies decreased oxygen extraction fraction (OEF). The reduction in OEF and CBF indicates the decrease in the cerebral metabolic rate of oxygen (CMRO_2_) and results in possible infarction [67].

To diagnose stroke, it is essential to examine the hemodynamic characteristics of the brain tissue. When a stroke occurs, it usually leads to hypoxia and damages the tissues in the brain [63,64]. With the specificity of hemoglobin detection using PAI, the resulting image from the pre-ischemic slice of an animal model shows that the distribution of HbO_2_ and HbR is symmetrical inside the brain. However, the distribution of HbR becomes asymmetrical in post-ischemic slices (Fig. 2) [65]. When using a photothrombotic ischemic stroke model, multi-spectral photoacoustic tracking demonstrates that the change percentage of HbO_2_ decreases and the change percentage of HbR increases obviously when a stroke occurs [66]. The impairments are observed by monitoring the capillary density, oxygen extraction fraction (OEF), and cerebral blood flow (CBF) after the onset of stroke in the targeted cerebral arteriole (Fig. 3) [67]. In addition, high-speed PAM is presented for blow flow redistribution monitoring in both cortical microenvironment and dynamic brain studies of mini-stroke mouse models, including cortical microhemorrhage and vessel occlusion, which usually occur in an aged brain [68]. This type of stroke does not have a severe impact on acute clinical stroke but can lead to cognitive decline and dementia. Imaging the hemodynamic response during stroke allows a better understanding of the mechanism of stroke, which opens a promising pathway to cortical microhemodynamics as a reference for cerebrovascular diseases and neurophysiological phenomena such as neuronal activity.

For the stroke treatment, PAI has been applied to image the cerebrovascular system to demonstrate the performance of the interventions. Currently, tPA is the only therapeutic drug approved by the Food and Drug Administration for acute ischemic stroke within the 4 h after the onset of the stroke [69,70], which is not available to over 96 % of the patients. To extend possible interventions, novel neuroprotective agents are widely investigated [26]. Using multi-parametric PAM, the intervention by sphingosine 1-phosphate (S1P), a bioactive metabolite of sphingolipids, has been revealed the potential for neuroprotection against cerebral hypoxia and ischemia [57]. Moreover, PAM has been combined with electrocorticography (ECoG) to acquire both hemoglobin information and brain activity signal on the research of therapeutic effects using integrated treatment modality of cathodal-transcranial direct current stimulation and peripheral sensory stimulation in a rat stroke model [71]. Combining with other detection modalities, PAI is capable of systematically and comprehensively investigating cerebral hemodynamics and metabolism of the effects of both pre-treatment and post-treatment in the brain.

Different from the stroke mostly happens in the adult, perinatal hypoxic-ischemic encephalopathy (HIE) is the main reason for the neonatal brain injury. Despite the causes of HIE and stroke are different, the researchers observed a similar hypoxic and ischemic phenomenon. PAI has been used to detect the changes of the cerebral hemodynamic in a neonatal rodent model [72]. Transcranial PAI also measured O_2_ saturation of the sagittal sinus in the hypoxia-ischemia model of neonatal piglets [73].

The representative researches about the PAI application on stroke until now have been summarized in Table 2. Most stroke models are induced in the middle cerebral artery of the brain. Apart from the middle cerebral artery of the brain, venous sinus of small animal has also been detected using PAI [74]. Besides, all works use small animal models for preclinical research. In these studies, mice with a lissencephalic brain are a prominent animal model. However, the lissencephalic brain differs from the gyrenchphalic brain. For example, spreading depolarization (SD), a phenomenon of near-complete neuronal depolarization with self-propagating waves [75], is widely referred to in neural conditions such as stroke [[76], [77], [78]]. SD not only occurs in the lissencephalic brain but also has been found complex patterns in the gyrenchphalic brain [79,80], and induced hemoglobin changes can be imaged by PAI [81]. This suggests that the deviation among various animal models needs to be taken into account when researching the stroke mechanism.

**Table 2:** PAI application of stroke in Mouse.

Year	System	Wavelength (nm)	Ultrasound (MHz)	Resolution	Stroke type	Induction	Refs.
2020	PAI^a^+ PAM + MRI	532, 680 - 950	5, 50	200 μm; 10 μm	Ischemic, hemorrhage	Photothrombosis and MCAO; silicone-coated monofilament	[82]
2019	PAT	532	5	A:1.62 mm	Stroke	Berberine	[83]
				L:180 um			
2018	PAM	532, 558	35	–	Ischemic	MCAO with filament	[57]
2018	MSOT^b^	–	–	–	Ischemic	565 nm LED	[66]
2018	MSOT^b^+MRI	680−980	5	–	Ischemic	tMCAO	[84,85]
2017	OR-PAM	532	50	A: ∼15 μm	Mini stroke	Phenylephrine	[68]
				L: ∼3 μm			
2017	ECoG + fPAM	808, 880	50	A:32 mm	Ischemic	Photothrombotic MACO^c^	[71]
				L:61 um			
2017	PAT	760, 840	5	A: 1 mm	Hemorrhage	Collagenase	[86]
				L: 120 μm			
2017	PAT	532, 750, 875	5	L: 180 um	Hemorrhage	Collagenase	[87]
2016	PAM	532, 559	–	–	Ischemic	Photothrombotic occlusion	[67]
2014	MSOT^b^	710, 850	–	–	Ischemic	MCAO	[65,88]
2013	ECoG + fPAM	560, 570	–	L: 65 μm	Ischemic	Photothrombotic stroke^c^	[89]
2011	OR-PAM	563, 570	50	L: 2.14 μm	Ischemic	tMCAO	[90]

PAI, photoacoustic imaging; PAM, photoacoustic microscopy; OR-PAM, optical-resolution photoacoustic microscopy; PAT, photoacoustic tomography; MRI, magnetic resonance imaging; MSOT, Multispectral optoacoustic tomography; ECoG-fPAM, electrocorticography functional photoacoustic microscopy; MCAO, middle cerebral artery occlusion; tMCAO, transient middle cerebral artery occlusion; L: lateral resolution; A: axial resolution Commercial equipment:

a Nexus 128 scanner, Endra Inc., Ann Arbor, MI.

b MSOT, iThera Medical Inc., Munich, Germany.

c Experiments in rats.

### 3.3 Diseases related to stroke using PAI

To understand the mechanism of stroke disease, it is important to show the influence of the other diseases such as atherosclerosis, cancer, and peripheral vascular functions and their potential impacts on the cerebrovascular system. Some of these diseases have been diagnosed using PAI, where the corresponding progress is summarized hereafter.

As a cerebrovascular disease, the occurrence of stroke can result from chronic diseases. For long-term monitoring of stroke disease, it is essential to monitor changes in the vascular system of other diseases. The main causes of stroke disease are vulnerable plaques and atherosclerosis, and their detection has been summarized in [91]. Diseases that cause blockage inside the blood vessel can lead to ischemic stroke. PAI can represent the blood clots in the mouse brain [92] and analyze the blood clot components under the blood background [93]. Abnormal cell shape diseases such as sickle cell disease can also be imaged using PAI [94]. Changes in the vascular wall can also lead to stroke. One typical disease is atherosclerosis caused by the accumulation of lipids and carbohydrates. Wavelengths around 800 nm [95,96] and 1200 nm [97,98] are usually used to image the lipid distribution for plaque detection in PAI and can be employed for atherosclerosis detection. The tumor is a type of disease in the vascular system that can also lead to stroke. Many studies on PAI for tumor imaging have shown good performance [[99], [100], [101], [102], [103]]. Furthermore, a peripheral vascular system disease can influence the cerebrovascular system and lead to the occurrence of stroke [104]. Abundant research has been performed to analyze the mechanism of the hemodynamics of cardiovascular disease using PAI [[105], [106], [107], [108], [109]]. Above all, PAI has been demonstrated for monitoring multiple hemodynamic and metabolic parameters in other diseases with high accuracy. Monitoring related diseases and understanding their mechanisms are also important for stroke prediction, diagnosis, and therapy.

Besides chronic diseases, unhealthy lifestyles and physical conditions increase the incidence of stroke [3,110]. The risk factors for stroke incidence include abnormal metabolism (high systolic blood pressure, high body mass index, high fasting plasma glucose, high total cholesterol, and low glomerular filtration rate), indisciplined behavior (smoking, poor diet, and low physical activity), poor environment quality (air pollution and lead exposure), and other undefined risk factors related to genetics. From the obesity mouse model research, PAI has shown that many parameters change within small arteries and veins between normal and obese samples [111]. Therefore, effective monitoring of risk factors is essential for stroke risk prediction in patients with diseases such as hypertension, atrial fibrillation, and diabetes mellitus. Physicians can then conduct appropriate interventions or preventions at the early stage before stroke occurrence.

### 3.4 Applications of PAI in both animal and human models

For the brain imaging, even the performance of PAI applied in large animals and human have been demonstrated, recently most applications of PAI have been conducted on small animals. We discuss in the following sections the main achievements in these models.

#### 3.4.1 Small animal as validation model

**Fig. 4:**
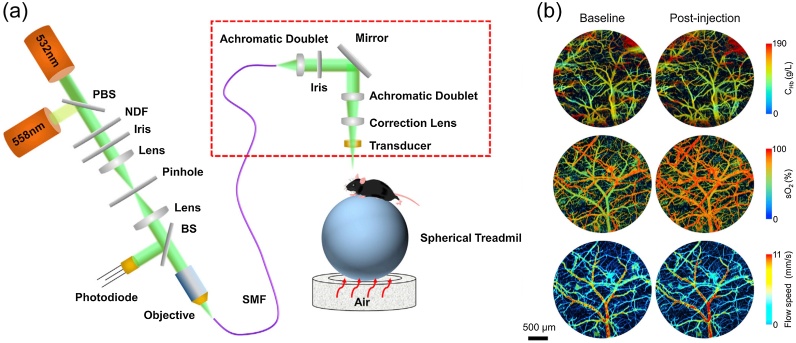
PAM of cerebrovascular reactivity in the obesity-induced awake mouse brain: (a) Schematic of the PAM with head-restrained for awake mouse brain; (b) Cerebrovascular images before and after obesity-induced change by the acetazolamide. Red dashed box is the scan head of PAM. PBS, polarizing beam splitter; NDF, neutral density filter; BS, beam sampler; SMF, single-mode fiber; C_Hb_, hemoglobin concentration; sO_2_, blood oxygenation (adapted from [111]).

Over the years, research groups generally use small animals such as mice and rats for stroke disease analyses. These groups focus on pathological mechanisms, imaging technique characteristics, and improvement. The small body size results in an easy manipulation of these animals during the experiments. Besides, the thin skulls of the mouse (∼0.2−0.3 mm) and rat (∼0.7−1 mm) make them less difficult for imaging. To have high resolution, PAM has been extensively used for brain imaging for small animals. Especially, small vessels (<40 μm) can be imaged using OR-PAM (Fig. 4) [111]. However, the resolution and penetration of PAI have an inverse relationship. For the deeper penetration, PACT with a wide imaging field has been used for brain imaging. And the structure of interior brain tissue located around 8 mm beneath the scalp has been visualized on a rat model with a scalp and intact skull using PACT [112].

#### 3.4.2 Large animal as validation model

The more the animal is large, the more its skull can be thick. For example, the thickness of the monkey’s skull is around 2−4 mm [113]. Recently, the PACT system was used in monkeys [[114], [115], [116]], piglets [73], and sheep [117]. Applications in large animals for stroke studies are rare, but several prior-art publications are describing PAI of cerebrovascular activities. Kirchner et al. researched the hemoglobin activity in the piglet’s head removed part of the skull [81]. Kang et al. used transcranial PA sensing to measure O_2_ saturation of the sagittal sinus in the hypoxia-ischemia model of neonatal piglets [73]. The outcome of the later study confirmed good measurements of O_2_ saturation ranging from 5 to 80 %. Jo et al. conducted PAI experiments to observe the functional responses in the motor cortex of awake monkey during forelimb movement [116]. Moreover, a non-invasive PAI study was compared with the invasive “gold standard” technique measured by a CO-Oximeter to monitor the cerebral venous blood oxygenation in superior sagittal sinus through the intact scalp of the sheep [117].

#### 3.4.3 Photoacoustic imaging in human subjects

So far, the PAI system has been used for human applications like breast, lymph node, prostate, systemic sclerosis, cardiovascular disease and graves disease [118]. According to the imaging specificity and depth, PAI is expected to be used for human brain studies. Due to the specific recognition of hemoglobin, stroke monitoring has become a typical application of PAI. Recently, results from the PAI technique applied to human brain monitoring have been published. Na et al. conducted functional human brain imaging on post-hemicraniectomy patients using three-dimensional PACT for the first time [119]. The system achieved recording the brain activities at a field of view (FOV) with a diameter of 10 cm, a spatial and temporal resolution of 350 μm/2 s, and a penetration depth of ∼2 cm. The progress on human brain imaging is a critical step toward stroke patients using PAI technology.

Due to the thickness of the human skull that is higher than the experimental animal ones, there are still large challenges to overcome in human brain imaging. Since the neonate’s skull is thinner than the adult, and the neonate’s fontanelle has not been closed [92], it has the forward potential of PAI for brain imaging. The use of PAI to monitor a neonate’s brain has been verified by animal experiments [73]. PAI of a human brain has been verified by an experiment on the phantom of a human skull [120]. The outlook of the PACT technique is the attempt of the human brain imaging through the skull *in vivo*.

### 3.5 PAI system development for human

Typical PAI-based equipment is smaller than CT and MRI systems, which is convenient for stroke application. In fact, a portable PAI system is a potentially fine solution for monitoring, diagnosis, and treatment in daily life. In this section, we analyze and compare three modalities of PAI system including hand-held system, wearable system, and intravascular PAI system. Those modalities can be potentially used in stroke applications for human beings, and prepare for the development of a clinical system.

#### 3.5.1 Hand-held detection systems

**Fig. 5:**
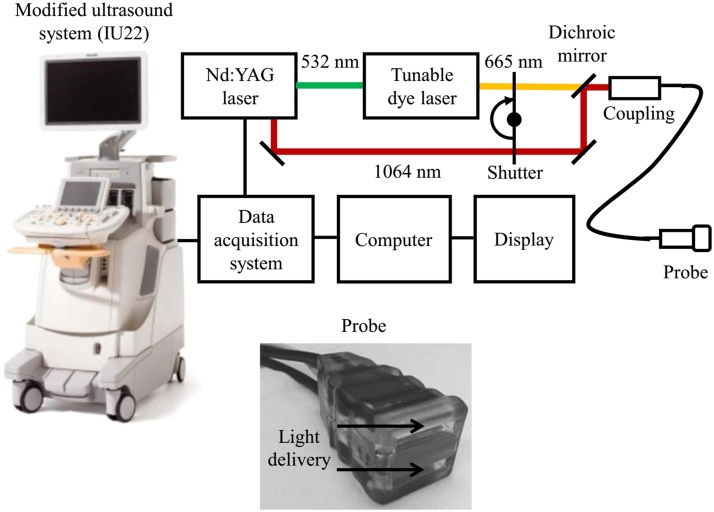
Block diagram of a dual-modality photoacoustic imaging system combined with ultrasound imaging [76].

Being a low-cost technique with non-ionizing radiation, PAI has the potential for clinical implementation as a hand-held imaging device like the present ultrasound imaging system. A hand-held PAI device has been used for many applications such as carotid artery [96], skin vascular, and melanoma [121]. A hand-held photoacoustic system is composed of a number of probes which can be designed and implemented as reported in [[122], [123], [124]]. Every probe includes a pulsed light source, and an ultrasound receiver, similar to the conventional ultrasound imaging system. In fact, a dual-modality photoacoustic imaging system can achieve both ultrasound and photoacoustic imaging functions (Fig. 5) [76].

**Fig. 6:**
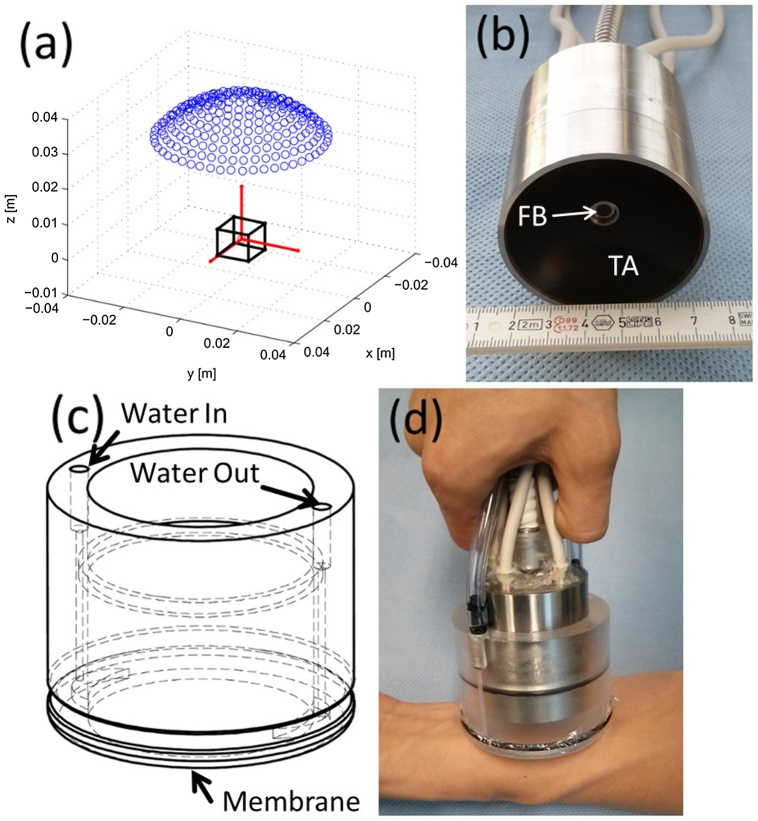
Three-dimensional hand-held PAI probe: (a) Layout of the piezoelectric elements drawn by blue dots and the region of the interest as the black cube, (b) Picture of the fiber bundle (FB) and ultrasound transducer array (TA), (c) Schematic of the water enclosing part, (d) Picture of the PA probe at the operation mode [125].

**Fig. 7:**
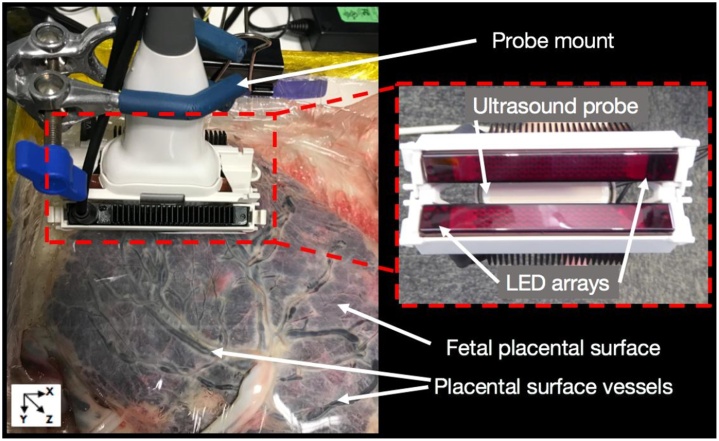
PAI system using light-emitting diode (LED) arrays mounted on the ultrasound probe for human placenta imaging [61].

The size of the light source of the PAI system can be reduced. Optical fiber is usually used in PAI probes, for example, a fiber bundle inserted through the center of a cylindrical cavity and surrounded by 256 piezoelectric elements of an ultrasound array is shown in (Fig. 6) [125]. Also, light sources can be laser or light-emitting diode (LED) (Fig. 7) [61]. Using LEDs with enough light eneryg instead of a laser source, can effectively reduce the size of the whole system. With the development of hand-held detection devices, PAI technique would soon be convenient for diagnosis purpose for several diseases not only for stroke.

#### 3.5.2 Wearable imaging systems

A PAI-based wearable device can be used to monitor the brain status. To construct images with good quality, a helmet grouping light sources and sensing interfaces is one of challenging design tasks. Present non-invasive, wearable brain monitoring systems are EEG and fNIRS, which use wearable caps to support data acquisition. In the case of fNIRS system, which needs incident and received signal coupling, the distance of the probes to the skin should be fixed through a cap to ensure high-quality signal acquisition [126]. In addition, the effect of hair on light transmission needs to be avoided. For a head-wearable photoacoustic detection device, the challenges are similar to those of an fNIRS device. First, the incident and received signals need to be coupled so that the focus points of the incident light and the ultrasound transducer are consistent. Second, the system needs to maintain stability in different environments such as motionless, acting but not moving, and acting and moving conditions. Third, user comfort also needs to be considered to avoid the influence of uncomfortable responses. According to the specific functional requirements, designing a suitable cap for different applications needs to be explored.

**Fig. 8:**
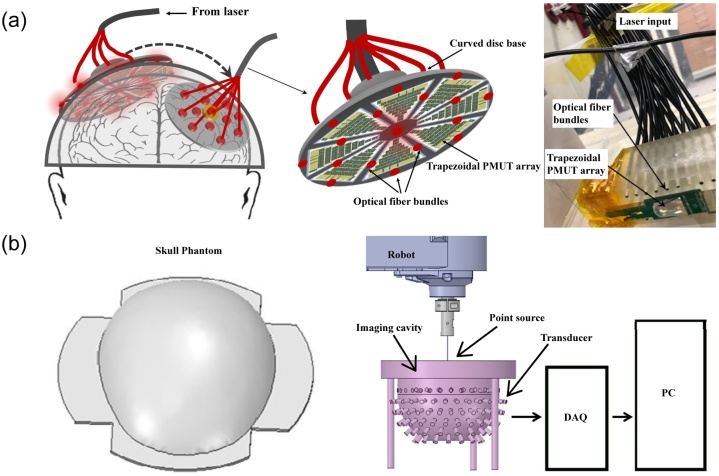
Schematic of PAI caps for human brain: (a) Spherical cap transducer arrays around the human head and the picture of a single piezoelectric micromachined ultrasound transducer array with light fiber bundles (adapted from [127]), (b) 3D models of the skull phantom and structure of PAI system (adapted from [128]).

Recently, wearable PAI systems have been introduced for human brain imaging. Dangi et al. proposed a device for neonatal brain imaging. Each working unit was installed individually with minimizing the size of back-end electronics. The PAI module was about 2-inch diameter and made up of a modular photoacoustic hat to cover the entire neonatal brain (Fig. 8a) [127]. Tavakolian et al. developed a neonatal brain imaging device built in a phantom skull model and customized PAI system (Fig. 8b) [128]. Notably, an imaging bowl was designed to hold the light sources, transducers, and the skull phantom in this research. The potential of the PAI for neonatal brain imaging has been demonstrated.

#### 3.5.3 Intravascular PAI systems

**Fig. 9:**
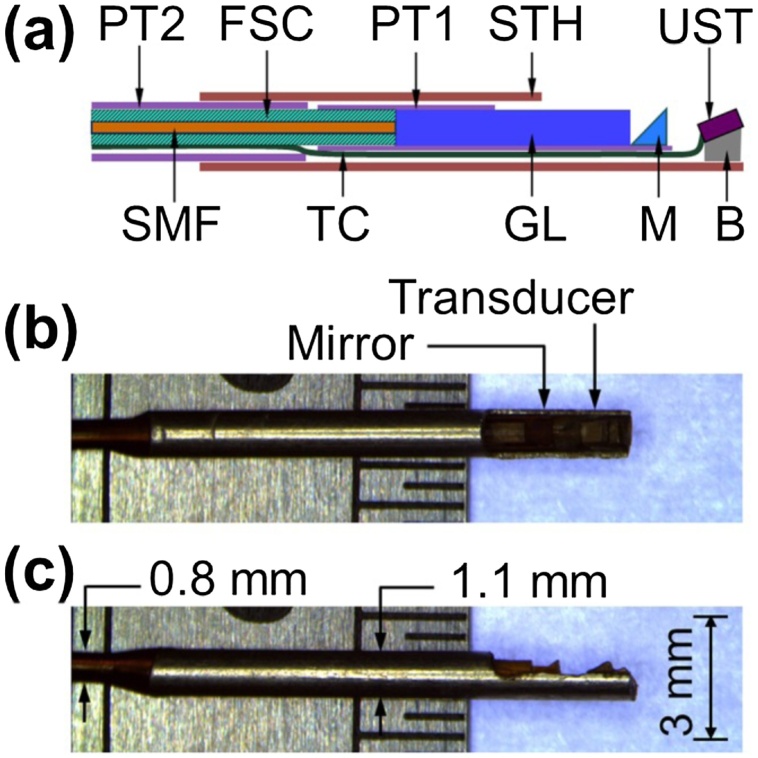
Schematic and micrograph of optical-resolution PAI catheter: (a) Schematic of the whole structure of the catheter, (b) Side view, and (c) Top view of the catheter micrographs. PT1, polyimide tube 1 with an 0.7 mm inner diameter; PT2, polyimide tube 2 with an 0.74 mm inner diameter; SMF, single-mode fiber; FSC, flexible stainless steel coil; TC, transducer cable; GL, gradient-index lens; STH, stainless steel tube housing; M, micro-prism; UST, ultrasonic transducer; B, metal base [129].

**Fig. 10:**
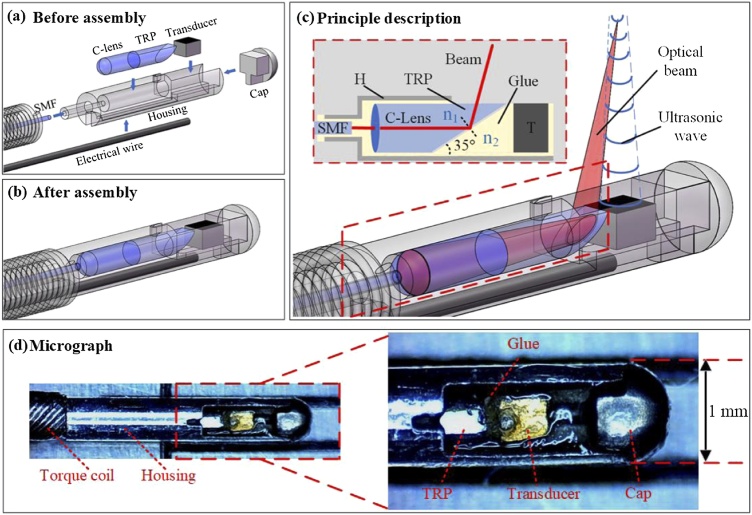
Schematic and photography of high-resolution intravascular PA endoscope (PAE) from design to assembly: (a) Schematic of the PAE before assembly, (b) Schematic of PAE after assembly, (c) Principle description of the system, (d) Micrograph of the PAE. SMF, single-mode fiber; H, housing; TRP, total-reflection prism; T, transducer [130].

In addition to non-invasive PAI systems, intravascular PAI technique has gradually been developed inside the blood vessel as an invasive imaging method, which is similar to the computed tomography angiography (CTA). The intravascular PAI method combines a light source and an ultrasound sensor is inserted in the cavity of a vessel (Fig. 9, Fig. 10). This intravascular PAI system has been used for arteriosclerosis application [129,130], but also can potentially be used for blood flow monitoring inside the vessels for stroke diseases. On the other hand, a PAI system based on a separate light source and an ultrasound reception module has been studied. The light source was inserted into the body using a catheter to provide internal illumination [98], which can improve the imaging depth due to optical diffuse inside the human tissue. In this case, the ultrasound detector was placed outside of the body. In the attempt of imaging the occlusion inside the brain, the pulsed laser light transferred by the optical fiber was inserted into the catheter to illuminate the occlusion tissue and the ultrasound transducer outside the tissue was applied to receive the generated PA signal [131]. The intravascular system can avoid optical or acoustic attenuation through the human body to achieve high-quality vascular imaging. With the improvement of the intravascular system, this modality potentially provides an important compensation in cerebrovascular imaging.

### 3.6 Multi-modality PAI

As a non-invasive detection method, PAI can be combined with other noninvasive detection methods for the daily monitoring of stroke patients. In this section, we describe different imaging techniques combined with PAI which are intended for various applications. These techniques are CT, MRI, and ultrasound for anatomical information, EEG for neural activity using the recorded electrical signals. Based on the achievements of the multi-modality combination, PAI can be eventually adopted for stroke application.

#### 3.6.1 PAI and ultrasound

**Fig. 11:**
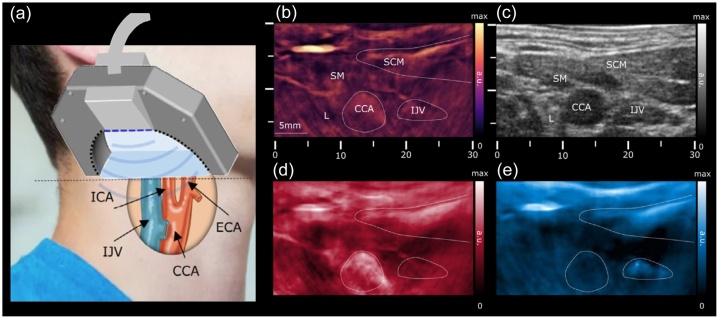
Carotid images using multi-mode (multi-spectral PAI, pulse-echo ultrasound, and color Doppler) PA image acquisition system: (a) Schematic diagram of the multi-mode imaging acquisition system with spectral unmixing PA images at seven wavelengths (700, 730, 760, 780, 800, 825, and 850 nm), (b) PA image at 800 nm of the main structures with marks, (c) Corresponding ultrasound image, (d) Spectral map of unmixed HbO_2_, (e) Corresponding spectral map of corresponding unmixed HbR. ICA, internal carotid artery; IJV, internal jugular vein; CCA, common carotid artery; ECA, external carotid artery; SM, strap muscle; SCM, sternocleidomastoid muscles [96].

Ultrasound imaging has been widely used in medical detection. Traditional ultrasound imaging has been combined with the PAI technique for dual-modality imaging toward *in vivo* biopsy of melanoma [29], morphological and physiological brain imaging [132] in the mouse experiments. Transcranial Doppler imaging is one application of brain imaging based on the ultrasound Doppler effect. However, ultrasound imaging has a low spatial resolution, and its specificity and sensitivity to blood are not high. Therefore, this technology still has obstacles to overcome. Multi-spectrum PAI, multi-modal pulse-echo ultrasound, and color Doppler imaging have been combined for hemodynamic imaging including blood flow and oxygen state in the carotid artery of a healthy person (Fig. 11) [96]. This work demonstrates that multi-modality has the potential to provide comprehensive information and can increase the accuracy of the assessment.

#### 3.6.2 PAI and MRI

**Fig. 12:**
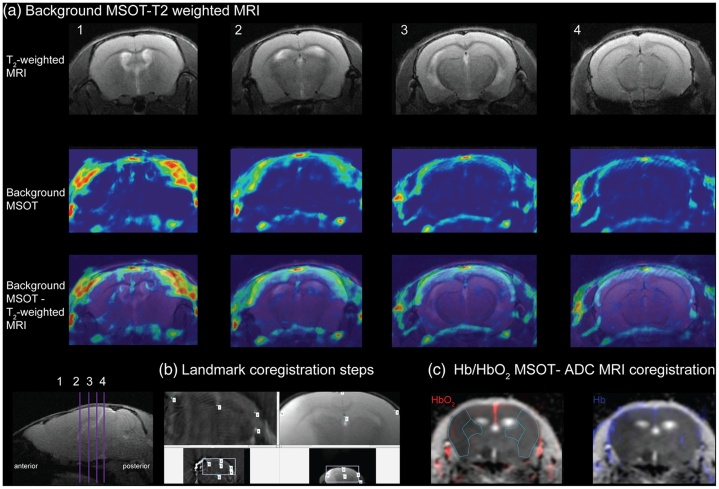
The procedure of MSOT-MRI data coregistration: (a) Coregistration of background MSOT and T2-weighted MRI images in the cross-sectional axial, (b) Landmark coregistration steps, (c) Coregistration result of Hb/HbO_2_ map by MSOT and apparent diffusion coefficient (ADC) map by MRI [85].

MRI can provide good structural information of the biological tissue or even small molecules. In the research of the cerebrovascular hypoxia and matrix-metalloproteinase (MMP) activity, multispectral PAI has been used to measure the oxygen metabolism (Hb, HbO_2_), MRI provides the structure of the MMP distribution (Fig. 12) [84,85]. Through the combination of two non-invasive imaging methods, the functional and structural information of different objects can be integrated. Once the application of the combination in the clinical stage, MRI can be done first to provide the structural information of human for PAI reconstruction and monitoring guidance.

#### 3.6.3 PAI and EEG

EEG is a non-invasive method for neural activity detection, where it acquires the corresponding electrical signal to the brain activities from the electrodes placed on the scalp. PAI has been combined with EEG to investigate the relationship between hemodynamics and neural activity [133]. Functional PAM has been studied with EEG to assess the neurovascular dynamics during the period of transient ischemic attack [134]. In addition, functional PAI can achieve direct neural mapping using specific contrast agents such as calcium indicators, which is comparable with the results from EEG [135]. An invasive method named electrocorticography has the same function as the EEG, has been used to couple with the functional PAM for the transient focal cerebral ischemia in rats [71,89]. Compared with EEG and ECoG, EEG has the obvious advantage that is a non-invasive method like the PAI. Thus, the combination of EEG and PAI has great potential in future applications in the human brain. With a better understanding of hemodynamics and neural activity, brain monitoring can provide potential information for stroke prediction and diagnosis.

#### 3.6.4 PAI and X-ray CT

**Fig. 13:**
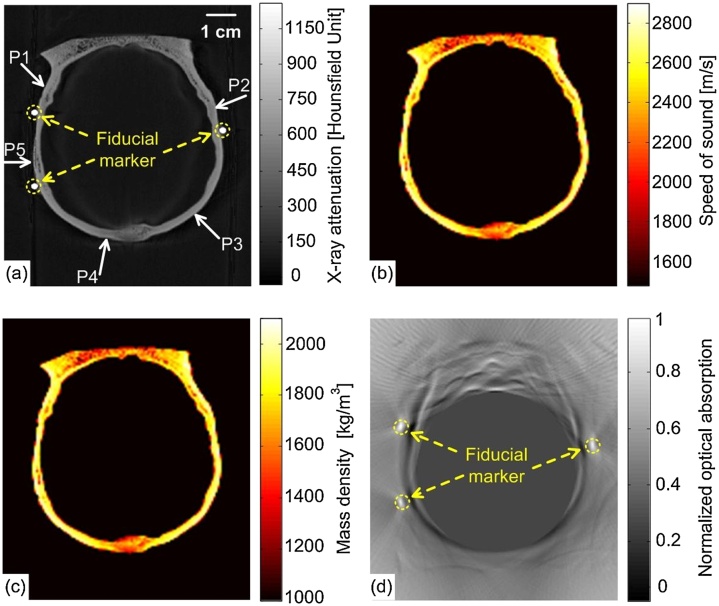
Imaging reconstruction of PAI based on X-ray CT data: (a) A two-dimensional slice of the X-ray CT of the skull labeled by fiducial markers through the PAI plane, (b) Speed-of-sound map derived from the X-ray CT data, (c) Density map derived from the X-ray CT data, (d) The PA image of the monkey head phantom reconstructed by the half-time algorithm [136].

Present researches regarding the combination of PAI and X-ray CT are mostly about the imaging reconstruction of transcranial PAI since the transcranial PAI for the human brain is a challenging work. According to the information of the skull provide by the X-ray CT image data, a time-reversal-based reconstruction algorithm has been developed for aberration correction for transcranial PAI [136] in the phantom and monkey head experimental studies (Fig. 13).

## 4 Challenges and prospects of PAI for stroke

**Fig. 14:**
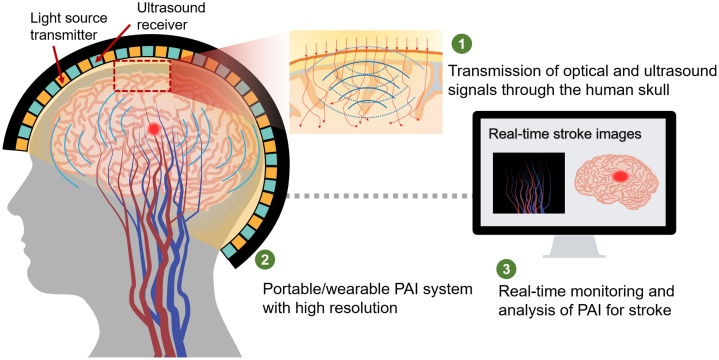
Challenges and prospects of PAI for human brain.

PAI is an important complementary approach to other imaging methods. With the development of PAI, this technique has gradually transitioned from the preclinical to clinical stages for applications such as breast and skin cancers. Also, it has excellent potential for stroke monitoring at different stages, from onset to rehabilitation. However, the present research progress of stroke monitoring mostly focuses on small animal models. With the obstacle of the human skull, PAI still has a long distance to go for clinical application in stroke monitoring. To achieve stroke imaging of the human brain, challenges and prospects shown in Fig. 14 can be explored as follow.

### 4.1 Transmission of optical and ultrasound signals through the human skull

As a non-invasive imaging method, transcranial PAI has enormous advantages for brain imaging. However, owing to the multilayer tissues of the head, optical and ultrasound signals are attenuated to different degrees. In particular, the skull significantly attenuates the propagation of both the optical and ultrasound signals. In fact, the light is strongly scattering at the depth beyond 1 mm [137].Therefore, it is difficult to transmit light into the skull and induce the PA effect in the deep region.

Even though the PA effect can be generated inside the brain, there is also a challenge to transmit the ultrasonic wave through the skull due to the mismatched acoustic impedance of the skull with surrounding tissues [113]. For the development of the PAI, modeling and simulation is a theoretic method to analyze the effect of the skull. The influence of the skull has been simulated using different methods like the Monte Carlo method and diffusion equation for optical propagation [138,139], and K-wave and Ray tracing for acoustic propagation [140]. During image reconstruction, the attenuation corrections based on the elastic wave equation [141], a layered back-projection method [142], and a vector space similarity mode [143] have been developed to get a good performance of the transcranial PAI.

Besides, medical images through CT and MRI have been processed to build 3D printing models [144] for image reconstruction and modeling simulation. Multi-modality imaging methods can not only help for transcranial PAI reconstruction but also provide the structural information of the human body. Moreover, deep learning algorithm has been used recently to overcome low image contrast and high structure loss in PAI reconstruction [145,146]. Also it has worked on calculating perceptually sensitive loss functions to virtually increase the maximum permissible exposure to enhance the PA images of the deep regions in the brain tissue [147].

### 4.2 Portable/wearable PAI system with high resolution

At present, a PAI system including a portable probe similar to that of ultrasound systems enables to scan organs such as the breast, or carotid artery. The size of the hand-held PA probe is still large [148], which is not convenient for daily monitoring. A portable system integrated the components in a small electronic board can reduce the size of the system. Nowadays, with the technological evolution, devices including amplifiers, digital-analog converters, controllers, and even sensors, can be combined with integrated circuits to decrease the size and cost of the system [149]. Developing an all-optical PAI system that replaces the piezoelectric scanning probes is another direction to provide wider bandwidth, finer spatial sampling [150]. The research on flexible materials can promote the design of emitters and sensors to better cover the detecting surface [151]. The laser source can be displaced by other smaller devices such as light-emitting diodes [152]. Moreover, the comfort of portable or wearable PA devices can be improved for daily health monitoring.

### 4.3 Real-time monitoring and analysis of PAI for stroke

Compared with CT and MRI medical equipment, the significant advantages of PAI are label-free and dynamic functional imaging with smaller equipment. Present, a wearable system like EEG and fNIRS has been used in stroke prediction [153], which is expected to be guidance on stroke monitoring and prediction. Although the present PAI system is limited for daily monitoring of human beings, the potential for body condition monitoring before, during, and after the onset of stroke should not be underestimated. The mechanism of stroke using PAI has only been studied in animals, but not in humans. In the future, more research on human beings must be explored. Using big data and artificial intelligence, stroke incidence prediction, preoperative diagnosis, and postoperative recovery can be realized based on PA images.

## 5 Conclusion

Photoacoustic imaging has been widely used in biomedical imaging applications over the past decades. In this review, we reported the current state of PAI focusing on its application to stroke diseases. Although most present stroke monitoring activities using PAI are limited to animal models, the advantages of PAI for stroke imaging cannot be ignored. Photoacoustic microscopy provides a good resolution and offers both structural and functional information of the cerebrovascular system. Furthermore, it can get the image of small vessels in small animals. Among the PAI technologies, PAM with high resolution can monitor the small vessels inside the mouse brain. To get deep and wide-field images, PACT has been used under the skull, which is expected to be applied in human brain imaging. The research status of the PAI application varying from small animals to human beings has been discussed. Despite the stroke imaging has not been used in the human due to the challenge of the brain skull, the imaging of skin and subcutaneous has been tested in the human body for the clinical applications. Besides, different imaging system structures are available for stroke application. Multi-modality PAI combined with other imaging techniques has been demonstrated to compensate present limitation of the PAI.

To promote the progress of the PAI for stroke monitoring, the system should overcome the problem of aberration from the human skull. Future work would focus on signal propagation inside the human brain and image reconstruction. Besides, the system structure should be optimized based on the requirement of the different imaging modalities including hand-held, wearable, and intravascular imaging systems. As a non-invasive imaging method, the modality of transcranial PAI has enormous advantages for brain imaging. With the improvement of the system, PAI can be used to better understand the mechanism of the stroke model for prediction, diagnosis, therapy from small animals to the human being. Hence, the PA technique is promising for stroke monitoring in clinical applications.

## Funding source

This work was supported by the funding from Westlake University (041030080118), Bright Dream Joint Institute for Intelligent Robotics (10318H991901), and Leading Innovative and Entrepreneur Team Introduction Program of Zhejiang (2020R01005).

## Declaration of Competing Interest

The authors declare that there are no conflicts of interest.
